# Unemployed individuals contact GPs more frequently but report lower satisfaction: results of the population-based DEGS1 and the GPCare-1 patient survey

**DOI:** 10.1038/s41598-022-10621-1

**Published:** 2022-04-23

**Authors:** N. Ikar, S. Sommer, M. Schmidt, C. Löwe, S. Kasten, B. Gavrilov, C. Hunzelar, F. Bockheim, J. Paños-Willuhn, L. Offenberg, M. Oberholz, B. Weltermann

**Affiliations:** Institute of General Practice and Family Medicine, University Hospital Bonn, University of Bonn, University of Bonn, Venusberg Campus 1, 53127 Bonn, Germany

**Keywords:** Public health, Health care, Risk factors

## Abstract

Unemployment is associated with a variety of adverse health-related outcomes, yet little data on primary care services for this risk group exist. Using data from two surveys, we analyzed the frequency of GP contacts and patients’ experiences with GPs comparing unemployed with employed individuals. Data of the *German Health Interview and Examination Survey for Adults* (DEGS1), a nationwide cross-sectional study (n = 8151), were analyzed regarding associations between employment status and the number of GP visits. The *General Practice Care-1 study* (GPCare-1), a cross-sectional questionnaire survey (n = 813), evaluated patients’ communication with their GP. Data were collected from June to August 2020 in 12 teaching practices affiliated with our university. The statistical analysis included individuals of working age (18–64 years old) (DEGS1 n = 5659, GPCare-1 n = 587). In both studies, working age subpopulations were analyzed (DEGS1: n = 5659 of 8151, GPCare-1: n = 587 of 813). In DEGS1, the prevalence of unemployment was 6.5% (n = 372). Unemployed individuals had more GP contacts in the last 12 months (4.50 vs. 2.86, p < 0.001). In the GPCare-1 study, unemployed individuals (6.6%, n = 39) were significantly less satisfied with GP communication: enough space in consultations (42.9% vs. 60.3%, p = 0.043), feeling comfortable to address sensitive topics (44.1% vs. 65.9%, p = 0.010), problems taken very seriously by GP (48.6% vs. 70.6%, p = 0.007). Yet, they were more willing to accept GPs’ help for psychosocial burdens (67.6% vs. 47.6%, p = 0.024). GPs should be aware that patients with unemployment wish more support to cope with their burdening situation.

## Introduction

Globally more than 192.7 million people (5.6%) were affected by unemployment in 2017^1^. According to the International Labour Organization a person is unemployed when fulfilling three criteria within the reference period: (i) the person is not in employment, (ii) the person is seeking work, and (iii) the person is available to take up work^1^. Unemployment can occur in all social classes regardless of educational level and age, and everyone can be directly or indirectly affected^2^.

In 2019, about 2.27 million people in Germany were registered as unemployed, corresponding to an annual unemployment rate of 5.0%^3^. Nearly two-thirds of all unemployed individuals were under the age of 50^4^. Of the unemployed, 0.72 million people had been unemployed for more than one year and are therefore classified as “long-term unemployed”^5^. In line with other studies, the 2009 GEDA study (German Health Update study) showed that unemployed individuals perceive immaterial losses as a burden^6^. In particular, the lack of contact with colleagues, the loss of social standing, and time structures are perceived as problematic^7,8^. Subsequent worsening of lifestyle, e.g., with increased smoking and alcohol consumption, is reported^9^.

Furthermore, unemployment is associated with various negative effects on physical and mental health outcomes, e.g., cardiovascular disease, type II diabetes and oncological diseases^2,10–12^. In the Survey on Health, Ageing and Retirement in Europe (SHARE; n = 11,462), 39% of the unemployed 50- to 64-year-olds reported poor health and 48% had a chronic disease which was significantly related to being unemployed^13^. A review of 237 cross-sectional and 87 longitudinal studies calculated a prevalence of 34% for mental disorders for unemployed compared to 16% for employed individuals^14^. The various negative health effects are amplified when individuals are long-term unemployed^15^.

General practitioners (GPs) are the first professional point of contact for health issues and have the potential to play an important role in providing comprehensive care to the unemployed as a high-risk group for adverse health outcomes^16^. A physician survey in Northern Germany revealed that GPs are confronted with the psychosocial burden of “poverty” (53.4%) and “work” (43.7%) at least three times per week^17^. Yet, little is known about the access of unemployed individuals to primary care and/or the quality of GP care from patients’ perspective. Using data of the population-based DEGS1 study, this study analyzed the frequency of GP visits during the last 12 months comparing individuals with and without unemployment experience. Applying the same stratification, data from the GPCare-1 patient survey addressed patients’ communication experiences with their GP and their willingness to accept professional support.

## Methods

### Study design

This study was based on data from two surveys:

1. The German Health Interview and Examination Survey for Adults (DEGS1) and.
2. The General Practice Care-1 patient survey (GPCare-1).

#### German health interview and examination survey for adults (DEGS1)

The DEGS survey, which is representative of the German population, was carried out by the Robert Koch Institute (RKI) as part of the German health monitoring system. Detailed information on the concept and design of DEGS1 is published elsewhere^18,19^. Survey-specific weighting factors are available to assure representativeness for the German population^18,19^. The DEGS1 data used for this analysis were kindly provided by the Robert Koch Institute.

##### Participants

The DEGS1 survey was conducted from November 2008 to December 2011. It included standardized computer-assisted personal interviews, self-administered questionnaires, as well as standardized examinations and additional medical tests. The target group was the German population aged between 18 and 79^18^. Data on 8151 participants are available. For this analysis, all individuals of working age (persons 18 to 64 years old) were analyzed (n = 5659).

##### DEGS1 measurements of socio-demographic characteristics

The following DEGS1 measurements of socio-demographic parameters were used for the analysis:

- *Age* in years.
- *Sex* male or female.
- *Marital status* married/in partnership or other.
- *Socioeconomic status (SES)* This was calculated using information on education, employment status and income, and was subsequently classified into the three groups low, middle and high SES (for details see^20^).
- Number of underage individuals in household.
- *Social support* Social support was determined by the Oslo 3-item Social Support Scale (Oslo-3) classified in three categories: low (3–8 points), middle (9–11 points), high (12–14 points)^21,22^.
- *Education and current employment status* All participants were asked for their highest level of education, their current profession, and whether they work full- or part-time. An additional multi-select question asked whether they were unemployed, in partial retirement, retirement/pension, retraining, internship, military/community service, housewife/husband, or none of these. To avoid any underestimation in our analysis, all individuals who marked unemployed were included in our analysis irrespective of additional answers. Thus, the group of unemployed included 9.4% who had marked housewife/husband, and 5.9% of respondents who had marked additional options.

##### DEGS1 measurements of health outcomes and GP contacts

The following items were used for the analysis:

- *Chronic stress in the last three months* This was measured by the Trier Inventory for Chronic Stress (TICS-SSCS)^23^ and was classified into three categories: low (0–11 points), middle (12–22 points) and high stress (22–28 points).
- *General health status* Participants’ answers on a five-point Likert scale were dichotomized (very good/good health versus mediocre/poor/very bad health).
- *Chronic disease* yes, no or don’t know.
- *History of physician-diagnosed depression* yes or no.
- *Depressive symptoms* Using the results of the 2-question patient health questionnaire (PHQ-2), a dichotomized score was calculated (score 0–2: has no depressive symptoms/scores 3 to 6: has depressive symptoms)^24^.
- *Current smoking* yes or no.
- *GP contacts* GP contacts were analyzed on the basis of answers to two questions: a) if a participant reported having a GP, and b) how often the participant visited the GP during the past 12 months.

#### General Practice Care-1 Study addressing patients’ communication with their GP (GPCare-1)

Data from the GPCare-1 patient survey were used to complement the DEGS1 findings on access to GP care with patients’ perception of their communication with GPs.

##### Practice and participant recruitment

Data were collected from June to August 2020 in 12 teaching practices affiliated with the University of Bonn. Based on publicly available statistics^25^, practices were selected to cover different socio-demographic regional characteristics (i.e., % unemployed, % of people in working age). The average unemployment rate of the practice regions was slightly higher than the nationwide comparative value (6.7% vs. 5.9%), but had a smaller range (3.6 to 9.2% vs. 3.6 to 11.2%). The average of people of working age in the practice regions was close to the national average (61.3% vs. 61.5%). All practices volunteered for the study. In the practice, the receptionist invited all adult patients who visited the practice during the recruitment period to participate. Patients were eligible if they had sufficient language skills and were mentally capable of completing the self-administered questionnaire in English, Arabic, Turkish, or German. Each patient received an information sheet detailing the study idea, the voluntary participation, and the anonymous approach. Patients were offered to mail the enveloped questionnaire to the institute or to put it into a study letter box in the office. For this analysis, the data of patients of working age (18 to 64 years old) were used (n = 587 of 813).

##### Measurements of the GPCare-1 study

To enable comparison, we designed a two-page questionnaire which could easily be completed by patients. Standard instruments (OSLO scale on social support, TICS inventory for chronic stress, and PHQ2 on depressive symptoms) were used identically to the DEGS-1, the other DEGS1-items were modified, mainly simplified.

##### GPCare-1 measurements of socio-demographic characteristics

- *Age* in years.
- *Sex* male, female or neutral.
- *Highest (professional) education* none; secondary school levels, vocational training, university degree, other. For the analyses, the answers were trichotomized: low education (no school education/secondary school up to 9th/up to 10th grade), middle education (high school (A-levels)/vocational school), and high education (university degree).
- *Current professional situation* employed; in vocational training, retirement/pension, housewife/househusband, self-employed, civil servant, traineeship, unemployed.
- *Spouse/partner* yes/no.
- *Caregiver* yes/no.
- *Parents and/or participant is foreign born* yes/no.
- Number of persons living in household.
- *Experience of job loss or unemployment* in the past, currently, or not applicable.
- *Experience of financial problems* in the past, currently, or not applicable.
- *Social support* Social support was determined and analyzed in the same way as the DEGS1.

##### GPCare-1 measurements of health outcomes and GP contacts

The following items were used for the analysis:

- *Chronic stress in the last three months* This was measured and analyzed by the Trier Inventory for Chronic Stress (TICS-SSCS) identically to the DEGS1^23^.
- *General health status* Participants’ answers on a six-point Likert scale were dichotomized (excellent/very good/good health versus mediocre/poor/very bad health).
- *Chronic disease* Participants were asked about frequent chronic diseases: coronary disease, stroke, hypertension, diabetes, depression, migraine, anxiety disorder, sleeping disturbance, chronic obstructive pulmonary disease, backpain, or none of these.
- *Depressive symptoms* These were measured with the short form PHQ-2 of the Patient Health Questionnaire (PHQ)^26^. A dichotomized score was calculated (score 0–2: has no depressive symptoms/scores 3 to 6: has depressive symptoms)^24^.
- *Time with current GP* Answer options included: less than 1 year, 1 to 2 years, 3 to 5 years, more than 5 years.
- *GP contacts* GP contacts were analyzed on the basis of answers to two questions: (a) if a participant reported having a GP, and (b) how often the participant visited the GP during the past 12 months.

As no validated screening tool for patient-physician communication on social problems is available in German, eight questions were constructed using existing questionnaires: the Patient Reactions Assessments (PRA-D)^27^, the Medical Interview Satisfaction Scale (MISS)^28^, the Patient Requests Form^29^, and the Patient-Doctor Relationship Questionnaire (PDRQ-9)^30^. The first four questions addressed patients’ experiences with their GP, the second four focused on patients’ communication preferences. All items used a five-point Likert-type scale (strongly disagree to strongly agree). The answer options were dichotomized (strongly agree/agree versus neutral/disagree/strongly disagree). The questionnaire was piloted by 40 individuals of the German general population with subsequent minor revisions of words and phrasing to ensure comprehension. The eight questionnaire items on patients’ communication experiences and preferences are detailed in Fig. 2.

### Statistical analyses

All statistical analyses were performed using the Statistical Package for Social Sciences (SPSS) 25.0 for Windows (IBM Corp., Armonk, NY, USA), with statistical significance set at p ≤ 0.05 (two-tailed). All analyses of the DEGS1 data were weighted using the survey-specific weighting factors provided by the Robert Koch Institute^18^. All measurements had missing rates of under 5.0%, with the exception of the number of underage individuals in the household (6.0% missing) and chronic disease (5.7% missing). Missing cases did not differ regarding gender, age or employment status, except a higher missing rate for chronic disease in individuals with a low socioeconomic background (low: 6.0%; middle 5.7%, high 4.0%; p = 0.038). Participants were excluded from analyses if data were missing for at least one relevant variable. Frequency distributions and descriptive estimates were used for the entire population aged between 18 and 64. Comparisons of the sub-populations of participants with and without a current unemployment status were conducted with Chi-square tests for categorical data and T-tests for numerical data. Multivariate analyses were used to estimate the effects of chronic disease, chronic stress, age, depressive symptoms, and history of unemployment on the number of GP contacts during the last 12 months; in this regard, all statistical requirements were reviewed and met. A statistical power analysis was performed for sample size estimation. Our proposed sample size of 4965 participants will be more than adequate for the main objective of this study.

Data analyses of the GPCare-1 study also encompassed Chi-square tests for categorical data and T-tests for numerical data, stratified by employed and unemployed patients. As n = 54 participants did not report their employment status, they were excluded from further analyses. Other missing rates of socio-demographic and health variables were under 5.0% with the exception of chronic stress (9.7%) and depressive symptoms (6.7%). Questions on communication experiences and preferences had missing rates of up to 10.0%. Missing cases did not differ with regard to key socio-demographic characteristics and were excluded from analyses if data were missing for at least one relevant variable.

### Ethics approval and consent to participate

The *DEGS1 survey* was consented with the Federal and State Commissioners for Data Protection and was approved by the Ethics Committee of Charité-Universitätsmedizin Berlin in September 2008 (No. EA2/047/08). Participants provided written informed consent before the interview and examination. In the *GPCare-1 study*, patients received written and verbal information on study procedures, confidentiality, and that participation was voluntary. Also, they were informed about the anonymous study approach and that the return of the questionnaire indicated their informed consent. The study was approved by the Ethics Committee of the Medical Faculty of the Rheinische Friedrich-Wilhelms-University Bonn in June 2020 (No. 215/20). The GPCare-1 study is registered in the German Clinical Trial Register (DRKS00022330).

Both studies were conducted in accordance with the 1964 Declaration of Helsinki and its later amendments or comparable ethical standards.

## Results

### DEGS1: Socio-demographics and health outcomes of DEGS1 participants of working age (18–64 years)

**Figure 1:**
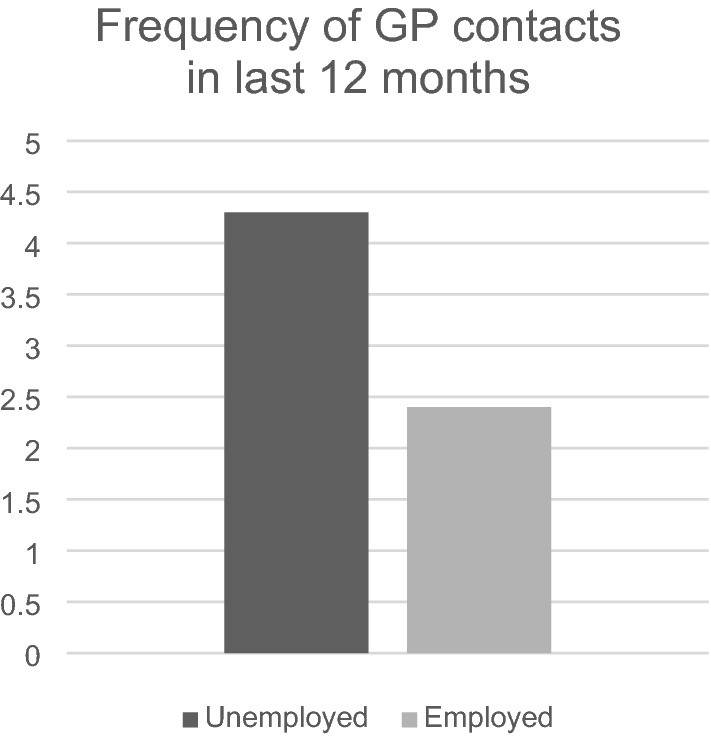
DEGS1 survey: frequency of GP contacts in the last 12 months (n = 5659).

Of the 5938 DEGS1 participants of working age, a total of 372 were unemployed at the time of the survey (“currently unemployed”). Comparing employed and unemployed individuals, there was no significant difference in gender distribution (unemployed: 47.1% vs. employed: 49.5% female) or age (mean = 42.34, SD ± 12.74 vs. mean = 41.53, SD ± 13.14). Unemployed individuals differed significantly from employed individuals regarding health-related characteristics, e.g., higher chronic stress (18.9% vs. 10.3%), a higher prevalence of having at least one chronic disease (36.7% vs. 23.4%), and a higher mean score on the PHQ-2 (mean = 1.47, SD ± 1.46 vs. mean = 0.98, SD ± 1.11). The groups did not differ regarding having a GP, but unemployed participants had visited their GP more frequently during the past 12 months (mean = 4.50, SD ± 7.51 vs. mean = 2.86, SD ± 3.99). For details see Table 1 and Fig. 1.

**Table 1:** DEGS1: Characteristics of participants (18–64 years): total and stratified by employment status (n = 5659).

Variables^a^	Total population (working age) (n = 5659)			Unemployed (n = 372)			Employed (n = 5287)			p-value
	n	%	95% CI	n	%	95% CI	n	%	95% CI	
Female gender	2987	49.3	47.7–50.9	189	47.1	40.6–53.7	2798	49.5	47.8–51.2	0.498
Age (mean, SD)	47.43 (16.69)		47.02–47.83	42.34 (12.74)		40.58–44.10	41.53 (13.14)		41.14–41.92	0.225
**SES**										
Low	795	17.2	15.8–18.7	175	54.0	47.1–50.7	620	14.5	13.1–16.0	< 0.001
Middle	3393	61.0	59.2–62.6	175	41.0	34.4–47.9	3218	62.4	60.7–64.2	
High	1468	21.8	20.1–23.7	22	5.0	2.7–9.1	1446	23.1	21.3–25.0	
Married/in partnership	3534	60.0	58.1–61.9	171	45.4	38.5–52.6	3363	61.1	59.2–63.0	< 0.001
**Children under 18**										
None	3648	64.9	63.0–66.8	268	70.6	63.8–76.6	3380	64.5	62.5–66.4	0.189
1–2	1626	32.3	30.5–34.2	80	27.6	21.7–34.5	1546	32.7	30.8–34.6	
3 +	128	2.8	2.2–3.4	5	1.8	0.6–4.8	123	2.8	2.3–3.5	
**Social support**										
Low	587	10.4	9.4–11.6	83	24.6	19.1–31.0	504	9.4	8.4–10.5	< 0.001
Middle	2797	49.8	48.1–51.5	197	48.2	41.5–54.9	2600	49.9	48.2–51.6	
High	2257	39.8	38.0–41.5	92	27.3	21.1–34.4	2165	40.7	38.9–42.5	
**Health**										
Smoking	1824	34.3	32.5–36.2	181	52.9	46.3–59.5	1643	32.9	31.2–34.8	< 0.001
**Chronic stress**										
Low	2914	51.2	49.5–52.9	144	36.8	31.0–43.0	2770	52.3	50.5–54.1	< 0.001
Middle	2119	37.9	36.2–39.6	158	44.3	37.4–51.5	1961	37.4	35.7–39.1	
High	603	10.9	9.9–12.0	65	18.9	13.5–25.7	538	10.3	9.3–11.4	
Good general health status	4480	79.7	78.4–81.0	220	58.4	52.0–64.5	4260	81.3	80.0–82.5	< 0.001
Chronic disease	1437	24.3	22.9–25.9	132	36.7	30.9–43.0	1305	23.4	21.9–25.0	< 0.001
Diagnosed depression	640	11.1	10.1–12.2	82	23.8	18.1–30.6	558	10.1	9.1–11.2	< 0.001
Depressive symptoms (mean, SD)	0.99 (1.15)		0.96–1.03	1.47 (1.46)		1.27–1.68	0.98 (1.11)		0.95–1.02	< 0.001
Has a GP	5088	89.1	87.6–90.3	341	90.3	84.7–93.9	4747	89.0	87.5–90.3	0.607
GP visits during 12 months (mean, SD)	3.19 (4.61)		3.02–3.36	4.50 (7.51)		3.28–5.73	2.86 (3.99)		2.69–3.02	< 0.001

^a^Results are based on weighted data of the DEGS1.

A multiple linear regression investigated associations between employment status and GP visits while controlling for covariates. The model included the variables age (over/under 50 years), employment, chronic stress, depressive symptoms, number of children, and chronic disease (yes/no). The variance of the model was R^2^ = 0.115 including n = 4965 participants, with no variables exceeding a variance inflation factor value of 1.6. Variables that were significantly associated with a higher number of GP visits were higher age, more depressive symptoms, and having a chronic disease. For details see Table 2.

**Table 2:** DEGS1: Regression model on factors influencing GP visits (past 12 months).

GP visits during the past 12 months (n = 4965)				
Parameter	B	SE	95% CI	p
Constant	2.519	0.432	1.666–3.372	< 0.001
Age < 50 years (ref.: 50 and older)	− 0.504	0.205	(− 0.908)–(− 0.100)	0.015
Employed (ref.: unemployed)	− 0.451	0.388	(− 1.216)–0.314	0.246
Chronic stress (TICS-SSCS)	0.015	0.010	(− 0.006)–0.035	0.153
Depressive symptoms (PHQ-2)	0.428	0.096	0.239–0.617	< 0.001
Number of children in household	− 0.093	0.064	(− 0.220)–0.034	0.150
Chronic disease (ref.: no)	2.607	0.234	2.146–3.068	< 0.001

### GPCare-1 study: Characteristics of the patient population of working age

A total of 813 patients participated in the GPCare-1 study. For this analysis, data of all individuals of working age were evaluated (n = 587). The patients’ average age was 43 years, the majority was female (60.6%, n = 350), and 7.3% (n = 39) were currently unemployed. For details see Table 3.

**Table 3:** GPCare-1: Characteristics of working-age patient population, stratified by employment status (n = 587).

	All patients < 65 age (n = 587)^a^		Unemployed (n = 39)		Employed (n = 494)		p-value
Variable	n	%	n	%	n	%	
Gender (female)	350	60.6	12	30.8	300	61.7	< 0.001
Age (mean, SD)	43.22	14.01	42.38	12.12	42.97	14.20	0.803
**Educational level**							0.062
Low	180	32.0	17	48.6	142	29.8	
Medium	237	42.1	10	28.6	206	43.3	
High	146	25.9	8	22.9	128	26.9	
In partnership	410	70.1	23	59.0	346	70.3	0.138
**Social support**							0.022
Low	120	21.3	12	35.3	96	20.1	
Middle	294	52.2	19	55.9	252	52.7	
High	149	26.5	3	8.8	130	27.2	
Chronic stress (mean, SD)	18.88	10.20	21.10	10.83	18.91	10.22	0.251
**Chronic stress**							0.412
Low	126	24.3	5	16.1	111	24.9	
Middle	211	40.7	12	38.7	179	40.1	
High	182	35.1	14	45.2	156	35.0	
Ever experienced job loss/unemployment	186	33.2	27	75.0	140	29.5	< 0.001
Currently burdened by job loss/unemployment	37	20.0	14	53.8	21	15.0	< 0.001
Ever experienced financial problems	205	36.5	22	57.9	169	35.5	0.006
Currently burdened by financial problems	52	25.4	9	40.9	38	22.5	0.059
**Health**							
Good general health	338	58.5	16	43.2	292	59.5	0.006
At least one chronic disease	349	62.4	30	78.9	291	61.7	0.034
Depression	103	18.4	9	23.7	83	17.5	0.344
Depressive symptoms (mean, SD)	1.85	1.66	2.60	1.87	1.85	1.67	0.011
**Years with current GP**							< 0.001
< 1 year	64	11.1	6	15.4	54	11.1	
1–2 years	71	12.3	12	30.8	55	11.3	
3–5 years	96	16.7	10	25.5	79	16.2	
> 5 years	345	59.9	11	28.2	299	61.4	
**Patients’ communication experiences with GPs**							
My doctor asks me about stress caused by personal strains	249	50.6	17	48.6	232	50.8	0.802
My doctor gives me enough space to describe personal strains	290	59.1	15	42.9	275	60.3	0.043
My doctor makes me feel comfortable talking about sensitive topics	311	64.4	15	44.1	296	65.9	0.010
I get the feeling that my doctor takes my problems very seriously	339	69.0	17	48.6	322	70.6	0.007
**Communication preferences**							
I prefer to overcome personal strain without help from my doctor	250	51.0	11	32.4	239	52.4	0.024
Discussing personal strains with my doctor makes me uncomfortable	169	34.7	12	35.3	157	34.7	0.940
I would prefer my doctor to ask me directly about personal strains	199	40.5	16	45.7	183	40.1	0.517
I would prefer the doctor to give me a questionnaire regarding my personal strains	134	27.2	17	48.6	117	25.5	0.003

^a^Statistical tests are based on n = 533 total participants, as n = 54 did not report their employment status.

Unemployed patients showed a higher prevalence of male gender (unemployed: 69.2% vs. employed: 38.3%, n = 186), low social support (35% vs. 20.1%), a lower prevalence of good general health (43.2%, vs. 59.5%), and had at least one chronic disease (78.9% vs. 61.7%). Also, they had a higher PHQ-2 score (mean = 2.6, SD ± 1.87 vs. mean = 1.85, SD ± 1.67). The majority of employed patients had been with their current GP for more than 5 years (61.4%, n = 299), as were 28.2% of the unemployed (n = 11).

### Patients’ communication experiences with their GP (GPCare-1)

**Figure 2:**
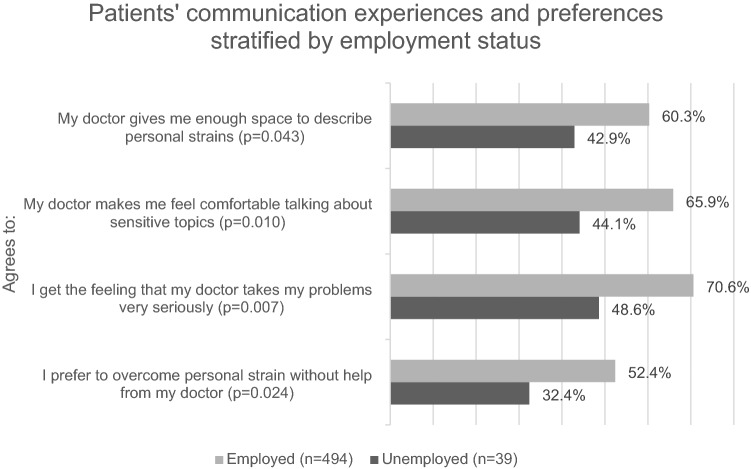
GPCare-1 study: patients’ communication experiences (n = 587) stratified by current employment status.

Of the patients of working age, 50.6% reported that their GP asks about personal problems and 69.0% felt taken seriously. Yet, unemployed patients reported significantly less satisfaction with care: “My doctor gives me enough space to describe personal strains” (unemployed: 42.9% vs. employed: 60.3%; p = 0.043), “My doctor makes me feel comfortable talking about sensitive issues” (44.1% vs. 65.9%; p = 0.010), “My doctor takes my problems very seriously” (48.6% vs. 70.6%; p = 0.007). While 51% of all patients preferred to overcome personal strains without a GP, the prevalence among the unemployed was significantly lower (32.4% vs. 52.4%; p = 0.024). For details see Fig. 2.

## Discussion

Based on the representative DEGS1 data, we showed a significantly higher frequency of GP contacts in the last 12 months by unemployed compared to employed individuals. This was strongly associated with chronic disease, higher age, and depressive symptoms. Despite more frequent contacts, GPCare-1 data show that while unemployed patients rated their communication with GPs as significantly less satisfactory than employed patients, they were more willing to accept GPs’ support to overcome their strain. To our knowledge, these findings on patients’ communication experiences are new and of relevance for primary care.

Our expectation was for younger people to have more issues arising from unemployment, as Hurrelmann et al. showed that unemployment in young families with children increases the risk of poverty^31^. However, even though our DEGS1 analysis showed that the prevalence of high chronic stress and depression were higher in unemployed than employed individuals, the number of children in the household does not influence the number of GP visits in our multivariate analysis. Nevertheless, unemployment has been shown to be associated with a higher prevalence of family conflicts^32^, and can affect the (mental) health of spouses and the family^33,34^. Thus, as pointed out by Harris and Harris, GP care should address not only the unemployed but also their next-of-kin as part of a setting-oriented approach.

The importance of communication and trust in patient-physician relationships to achieve the best subjective and objective health outcomes is well documented^35,36^. While various risk groups for poor outcomes are addressed in primary care research (e.g., patients who have experienced sexual abuse, drug dependency, seniors, migrants), the unemployed have been poorly addressed thus far. A PubMed search performed in 12/2021 yielded over 11,000 citations for the keywords “unemployment and health”, but only four citations when combining “unemployment and health and primary care”. In two of these four publications, the Australian GP scientists M. and E. Harris raise the important issue of GPs’ management of patients who become unemployed^37^. They address the various challenges involved, namely psychological and physical problems including sleep disturbances, anxiety, depression, and worsening of cardiovascular risk factors. Also, they describe the need to apply effective approaches in GP practices where good communication is critical, such as cognitive behavioral techniques, motivational counselling, and goal setting. GPs’ support should include appropriate medical certificates, advocacy as well as social support to “help redress the loss of the personal and social ‘vitamins’ of work”^37^.

A review by Fong Ha et al. concluded that communication is essential for a therapeutic doctor-patient relationship^38^. Especially in non-gatekeeper systems, patients feel a great need for communication about their psychosocial burdens^39^. Our GPCare-1 study from the German non-gatekeeper system is in line with this finding for the unemployed: only 32% are not willing to accept GP support. Interestingly, both unemployed and employed patients reported that their GP asks about personal strains, yet the unemployed reported significantly less frequently that they had enough space in consultations, felt comfortable to address personal topics, and that their GP takes their problems very seriously. These findings call for increased awareness on behalf of physicians and show the need to provide them with practical guidelines on how to communicate with and care for unemployed patients. The need for such approaches is emphasized by our results from the DEGS1 and by other studies^2,10–12^ which showed a higher prevalence of adverse health-related outcomes in the unemployed compared to the employed. In Germany, an integrated approach of social welfare and employment offices has been implemented to improve person-centered addressing and commitment to the unemployed, but systematic approach to address health issues has not been integrated. In this system, it could be beneficial to implement the recommendation made by Harris and Harris in 2009 to provide comprehensive care addressing psychological, health, and social issues by means of an interdisciplinary approach with family physicians, social workers, and psychological counsellors^16^. The importance of such an approach is emphasized by the fact that unemployed individuals report significantly less social support than those in employment. Thus, future family medicine research should consider a comprehensive view on the unemployed as an important target group^37^.

### Strengths and limitations

Our analysis used data from DEGS1, a large representative study which included all social classes and age groups. The GPCare-1 questionnaire was translated into different languages to reach patients from different ethnic groups in GP practices. The GPCare-1 study focusses on patients’ psychosocial strain and used an approach novel to the German health care context. Due to the cross-sectional design of both studies no causal relationships can be determined. The COVID pandemic, which changed practice routines and patients’ attendance through hygiene concepts, precautionary measures, and infrastructural adaptation, might have influenced the participation and results of the GPCare-1 study. This may have disproportionately affected high-risk and/or careful patients, who were more likely to avoid additional contacts. In addition, patients with acute complaints may not have been inclined to or able to participate. While the data of the DEGS1 on the frequency of GP visits are representative, the GPCare-1 data are not and sampling biases cannot be excluded. Due to the limited number of 39 unemployed cases in the GPCare-1 study, results from both studies cannot be directly compared. As the survey took place during a lockdown in the context of the COVID pandemic, practices were too busy to provide information on the number of patients eligible and recruited; participation rates could therefore not be calculated. We cannot exclude minor oversampling of unemployed individuals within the limited sampling time of the GPCare-1 study; however, the figure of 6.6% unemployed is within the national range. In both studies, the number of unemployed participants was low (DEGS1: n = 372, GPCare-1; n = 39) due to the population sampled, which limits possible interpretations of the results. The limited number of cases was taken into account in the selection of the statistical analyses.

## Conclusion

Our study focusses on currently unemployed individuals as a risk group for adverse health outcomes. The result of higher GP attendance by the unemployed, but more unsatisfactory communication is equally reassuring and challenging for German primary care. GPs should be aware of patients with unemployment experience as a risk group for adverse outcomes and should be prepared to intensify communication to address their needs. Our study highlights the openness of unemployed patients to accept professional help as an important resource.

## Data Availability

The DEGS1 dataset underlying this article was provided by the ‘Health Monitoring’ Research Data Centre at the Robert Koch Institute (RKI), which is accredited by the German Data Forum according to uniform and transparent standards (http://www.ratswd.de/en/data-infrastructure/rdc). Data are accessible on application to interested scientists for anonymous scientific secondary analyses. Detailed information on access, application forms, and guidelines can be obtained from datennutzung@rki.de. The dataset of the GPCare-1 patient study will be shared on reasonable request to the Institute of General Practice and Family Medicine of the University of Bonn, Germany.
